# Richard Bright's Reports of Medical Cases (1827): A Sesquicentennial Note

**Published:** 1978

**Authors:** Jeffrey Boss

**Affiliations:** Department of Physiology, Medical School, University Walk, Bristol, BS8 1TD


### Richard Bright's Reports of Medical Cases (1827): A sesquicentennial note

**Jeffrey Boss**
Department of Physiology, Medical School, University Walk, Bristol, BS8 1TD

The purpose of this paper is to note the importance of Richard Bright's *Reports of Medical Cases*[1] for renal physiology, and to ask what the *Reports* may teach us about the relation between physiology and clinical medicine not only in its own time but also to-day, a century and a half after publication. The greater part and central matter of the *Reports* are descriptions of twenty-three cases of renal disease.

### BIOGRAPHICAL NOTE

Richard Bright was born on the 28th September 1789, at 28 Queen Square, Bristol[2] in a house which survived the riots of 1831 and still stands in its terrace, the shell porch directed to the tree-shaded grass where Bristol office workers to-day eat their packed lunches[3]. In 1808 Bright entered Edinburgh University, becoming a student at Guy's Hospital in 1810, in which year he visited and explored Iceland. In 1811 he published a paper on the geology of Bristol[4], but this seems not to have interfered with his medical studies, since he wrote on bleeding, gangrene and erysipelas in 1812 and 1813, graduating M.D. in the latter year[5]. During 1814 and 1815 Bright toured on the Continent, spending some months in Hungary[6] where his visit and exchange of ideas with physicians are still regarded warmly. In 1815 Bright read a communication on volcanic formations in Hungary, and in the following year received the licence of the Royal College of Physicians and an appointment to the London Fever Hospital. In 1818 he travelled again, this time in Italy, Germany and France. Two years later he became assistant physician at Guy's, and in 1821 was elected a Fellow of the Royal Society. From 1822 to 1825 Bright lectured on botany and materia medica at Guy's, where he was appointed a full physician in 1824. In the following year medicine became the field of his lectures. Bright was admitted to fellowship of the Royal College of Physicians in 1832. He resigned from Guy's in 1843, and died in 1858.

It is from the years after Bright's appointment as full physician at Guy's that we have his major contributions to medicine, including renal medicine. Following his twenty-three cases, published in 1827 and already referred to, Bright gave the Gulstonian Lecture for 1833, reporting his observations on urine; in 1836 he published his final and most extensive series on observations on renal disease. In the course of this work Bright correlated clinical signs, especially oedema, with renal morbid anatomy, and albuminuria with both. The work was crowned by Owen Rees, Bright's assistant, who in 1850 published his *Diseases of the Kidneys*, in which a raised concentration of urea in the blood was reported as a feature to include in the constellation of clinical and post-mortem characteristics of renal disease. It is hardly surprising that the group of conditions of which this constellation is diagnostic came to be called Bright's disease, a name used well into this century and still familiar to-day.

### BRIGHT'S ACHIEVEMENT

The clinical recognition of renal disease made the distinction between cardiac and renal oedema possible, and hence the more effective use of digitalis in the former. The cardiac patient is an indirect beneficiary of Bright's researches; the patients with Bright's disease also have some benefit, for he found methods of improving and even apparently curing sufferers from the condition he described. Nevertheless, even now, we do not cure Bright's disease but, in the last resort, replace kidneys.

In the correlation of morbid anatomy with clinical observations, Bright's studies were and are outstanding. This approach to the characterisation of disease reached its full flowering in the Vienna school of Rokitansky; Bright can now be seen, in retrospect, as its worthy forerunner.

It is in physiology that we can see Bright's observations and reasoning to have been nodal in the network of paths of discovery. Oedema is the retention in the body of a normal constituent of urine, water. Albuminuria is the loss in the urine of a substance normally retained in the body. The regular occurrence of both with visibly abnormal kidneys indicated these organs to be a gateway controlling the loss and retention of components of the body. This concept, so obvious to us, was not obvious either to physicians or to physiologists before Bright. After him the central question in renal physiology becomes what it is now: how, in the formation of urine, are the constituents of blood reproportioned and controlled in amount? Bowman[7] and Henle established the histological knowledge enabling the question to be broken down into its references to Malpighian corpuscle and tubules respectively, the parts played by these becoming clearer with Richards's micro-puncture studies in our own century. Meanwhile, Claude Bernard's work raised the question as to how variations in the proportions and amounts of substances lost in the urine might be varied so as to maintain constant the composition of the *milieu intérieur.*

Thus Bright's study of the kidney marks the beginning of renal physiology as a science with a continuing and developing intellectual structure. Once through the door which Bright opened physiologists could explore renal function in more than one direction.

### THE CLINICIAN AS PHYSIOLOGIST

So it was that a physician in clinical practice made a major contribution to physiology and, in this respect, we may ask what 1827 has to teach 1977. How does Bright, in the Quaker phrase, speak to our condition?

There are five points to be made in clearing the ground for an answer. The first is that Bright did not, as William Harvey had done, practice medicine and physiology as separate activities. Secondly, the investigations not directly of the living patient, namely of the cadaver, urine and (in later work) blood, are those commonly considered integral with clinical practice, even by the least technological of doctors and Bright used no other methods away from the bedside. Next, we may note that what Bright did for his patients was in no way distorted to accommodate his desire to investigate. Fourthly, and consequent upon the previous points, Bright's scientific enquiry and clinical practice were integral. Finally, we may see that his enquiry and medical care positively reinforced each other. A reading of the case histories shows concerned daily care for each patient, and how bedside observations of scientific importance arose directly from that care, including required therapeutic action. Conversely, Bright's scientific drive quite evidently heightened his sensitivity in optimising individual treatment. So what then do we learn from *Reports on Medical Cases* for our own science and practice after just one-and-a-half centuries? *Verbum sapienti satis.*

### NOTES

1. *Reports of medical cases selected with a view of illustrating the symptoms and cure of diseases by a reference to morbid anatomy*, by Richard Bright, M.D., F.R.S. & C., London (Longman et al) 2 vols. in 3, 1827–31. (Reprinted in A. A. Osman (ed.), *Original papers of Richard Bright on renal disease,* Oxford University Press, 1937); (the reprint loses some of the quality of Bright's coloured engravings showing the cut surfaces and outer aspects of diseased kidneys).
2. The house is shown to be that of Lowbridge Bright, Esq., Merchant, in W. Routh's *The Bristol and Bath Directory,* Bristol, 1787, and the family name recurs at this address in *Matthew's New Bristol Directory for the year 1795.*
3. The house, now in commercial use, has no visible indication that it is Richard Bright's birthplace.
4. After leaving Bristol, Bright kept in touch with his native city. The Medical Library of the University of Bristol holds a copy of the *Reports of medical cases* (as in n.l, *supra*), inscribed as follows:
   - Vol. 1: 'The Medical Society of Bristol from the Author', in Bright's handwriting. Two book labels, not in his writing: 'No 763 Bristol Medical Library. Presented by the Author December 1835' and 'Presented by Dr. Bright to the Bristol Medical Library Dec r 1835'.
   - Vol. 2: pts. 1 and 2: 'From the Author', in Bright's handwriting, in each volume.
   - Vol. 2: pt. 1 only: Book label, 'Presented by Dr. Bright to the Bristol Medical Library Dec r 1835'.
5. Where no other work is cited, the events of Bright's life noted here are drawn from the following secondary sources. (i) J. F. Payne, in *Dictionary of National Biography, London,* 1908, *s.v.* Bright, Richard. (ii) E. A. Underwood, in *Chamber's Encyclopaedia,* last edition, *s.v.* Bright, Richard. (iii) R. T. Williamson, *English physicians of the past,* Newcastle-upon-Tyne (Reid), 1923; pp. 87–95. (iv) Sir William Hale-White, *Great doctors of the nineteenth century,* London (Arnold) 1935; pp. 63–84. (v) Unsigned article on *Bright, Richard* in *Encyclopaedia Britannica,* 11th edition.

NOTES *continued on page 18*

*continued from page 6*

**Richard Bright's Reports of Medical Cases (1827): A sesquicentennial note**

6. Cf Richard Bright, *Travels from Vienna through lower Hungary – 1814*, London, 1818. This is available for public consultation in the Central Library of the City of Bristol. Many aspects of Austria and Hungary and of life in them are noted, and the author's drawings give valuable additional information. Bright who was a linguist as well as a keen traveller, includes a vocabulary of English to Hungarian and to Hungarian Romany. The book was used as a guide to Hungary for much of the nineteenth century, and has a good name among Hungarians themselves.
7. William Bowman, *Collected papers*, ed. J. Burdon-Sanderson and J. W. Hulke, London (Harrison), 1892. Bowman's study of the kidney was published in 1842.

